# Effects of Elevated Glucose on Bacterial Respiratory Infections in Cystic Fibrosis and Chronic Airway Diseases

**DOI:** 10.3390/ijms26125597

**Published:** 2025-06-11

**Authors:** Emily M. Hughes, Megan R. Kiedrowski

**Affiliations:** Division of Pulmonary, Allergy and Critical Care, Department of Medicine, Heersink School of Medicine, The University of Alabama at Birmingham, Birmingham, AL 35294, USA; ehughes2@uab.edu

**Keywords:** diabetes, hyperglycemia, cystic fibrosis, cystic fibrosis-related diabetes, pneumonia, bacterial pathogens

## Abstract

People with diabetes are at increased risk of developing lung infections and have more severe complications. However, the link between these risks and outcomes is unknown. These trends are also seen in people with chronic lung diseases, including cystic fibrosis (CF); however, less is known about the underlying mechanism of disease in these cases. Traditional CF bacterial pathogens are often associated with worse disease outcomes in non-CF individuals with diabetes or hyperglycemia who have other acute or chronic airway disease, yet how diabetes and hyperglycemia further compound chronic CF infections is less clear. In this review, we focus on what has been observed clinically regarding bacterial respiratory infections and diabetes, and we discuss model systems used to study these relationships. We also review what is known about the role of diabetes in chronic CF lung disease and how information gleaned from the general population can inform future research directions in the new era of highly effective modulator therapies for CF.

## 1. Introduction

It has been well documented that hyperglycemia and diabetes are associated with decreased lung function [1,2,3,4,5]. However, the mechanism by which this decline occurs is still unknown. Diabetes is the result of chronic hyperglycemia caused by insulin dysregulation. In people with Type 1 diabetes (T1D), this dysregulation is caused by the loss of insulin-producing pancreatic beta cells due autoimmune dysfunction [6]. In Type 2 diabetes (T2D), insulin is still produced, but the lack of proper cellular responses to insulin results in high blood sugar [7]. Studies across multiple countries have shown that people with chronic diabetes mellitus, including T1D and T2D, are at increased risk for developing pneumonia (adjusted odds ratio, T1D = 1.42 [95% Cl, 0.96–2.08], T2D = 1.32 [95% Cl, 1.13–1.53]) and have worse outcomes than those who do not have diabetes, including in diagnosed and undiagnosed populations (hazard ratio of 2.00 [95% Cl 1.34–2.98]) [8,9,10,11,12,13,14,15]. Despite this knowledge, it has not been clearly defined why these populations are more at risk, nor has it been well documented what the underlying causes of pneumonia are, with respect to bacterial and viral pathogens.

In people with chronic lung diseases, like chronic obstructive pulmonary disease (COPD) and cystic fibrosis (CF), worse outcomes are commonly observed when diabetes is a confounding factor, including increase in frequency and number of exacerbations [16,17,18,19,20,21,22]. Over the course of a year, individuals with COPD and diabetes had a mean exacerbation frequency of 2.4 ± 0.8, while those with COPD alone had a lower exacerbation frequency of 0.68 ± 0.5 [22]. In CF populations, one study found 36.4% of people with CF and diabetes had three or more exacerbations in a year, compared to only 9.5% of people with CF but no diabetes [23]. Furthermore, up to 30% of European adults with CF and 50% of adults in the United States with CF will experience a CF-specific type of diabetes referred to as CF-related diabetes (CFRD), a condition that arises due to pancreatic damage over time from a lack of functional cystic fibrosis transmembrane conductance regulator (CFTR) activity in the pancreas [17,24,25]. In this review, we focus on what is known regarding elevated airway glucose in CF and chronic airway diseases and how knowledge obtained from studies of elevated glucose in acute respiratory disease can identify potential similarities and areas of future interest to investigate regarding chronic respiratory diseases.

## 2. Regulation of Airway Glucose Homeostasis

One commonality between individuals with diabetes and individuals with chronic lung disease is an increase in glucose availability in the lungs. In non-diabetic and non-disease states, the healthy lung epithelium tightly regulates glucose homeostasis in the airways. Regulation of paracellular and transcellular glucose transport keeps free glucose in the non-diseased airway at concentrations well below 1 mM in the airway lumen. However, hyperglycemia, chronic high blood glucose conditions like diabetes, and airway disease can result in elevated glucose concentrations detectable in the lungs, as summarized in Table 1. Hyperglycemia in otherwise healthy individuals can cause airway glucose concentrations to increase up to 0.51–1.89 mM [26] It has been hypothesized that glucose restriction in the non-diseased airways is one factor that helps to control bacterial growth in the airway by limiting nutritional availability for microbes that colonize the respiratory tract or preventing invasion by opportunistic pathogens [27].

Glucose homeostasis in the airways is controlled by innate epithelial barriers and by cellular transporters. Paracellular transport of glucose via tight junctions facilitates the movement of glucose from the bloodstream to the airways. Intact tight junctions prevent leakage of glucose and other blood components into the airway space, whereas infection and airway diseases may compromise epithelial barrier integrity. It has been demonstrated that proinflammatory mediators TNF-α and INF-γ, as well as bacterial lipopolysaccharides (LPS), can disrupt glucose homoeostasis in the airways [28]. This most likely occurs through the disruption of tight junctions [29,30].

To date, 14 different genes encoding cellular glucose transporters (GLUTs) have been identified, and recent work has identified 12 GLUTs in the human airways. These transporters are responsible for passively transporting glucose out of the lumen and into airway epithelial cells [31]. GLUTs in the airways have been identified in primary human airway tissue cultured ex vivo. mRNA transcripts of GLUTs 1, 3, 5, 6, 8, 9, 11, 12, and 13 were detected, and GLUTs 4 and 10 have been identified by both mRNA and protein, while, to date, only GLUT 2 protein has been identified and reported [31]. Paracellular glucose transport and transport via GLUTs only occurs when there is a glucose concentration gradient present. Sodium-coupled glucose transporters (SGLTs) found in the small airways are active glucose transporters and are, therefore, able to move glucose from areas of lower concentration into areas of higher concentration. The proper function of GLUTs and SGLTs is essential to maintaining low glucose concentrations in the airway surface liquid (ASL) [32]. However, it was recently shown that hyperglycemia dysregulated GLUT protein expression levels in the lungs in a Type 1 diabetes mouse model [33].

**Table 1:** Glucose availability in the lungs measured in healthy and chronic airway disease states.

Disease State	Sampling Method	Glucose [mM] Measured
No disease	Breath Condensate [26] Sputum [34]	0.40 ± 0.24 ≤0.50
Diabetes	Breath Condensate [26]	1.20 ± 0.69
Cystic Fibrosis	Breath Condensate [26] Sputum [35] Sputum [36]	2.04 ± 1.14 0–65.00 3.0
CFRD	Breath Condensate [26] Sputum [35]	4.00 ± 2.07 0–15.00
COPD	Sputum [34]	Average 0.75 Up to 2.50

## 3. Measurement of Airway Glucose

Measuring glucose concentrations in the airway is a challenging task compared to measuring blood glucose levels. Airway glucose can be approximated by measuring glucose present in mucus and breath samples. Depending on the sensitivity of the technique used, glucose in the airways of healthy individuals is often too low to be measured, resulting in studies that have quantified glucose availability as detectable or undetectable [37]. The breath condensate method is one technique that uses mass spectrometry to measure glucose concentrations in breath samples. To obtain breath condensate measurements, repeat water mouthwashes can first be performed to ensure breath glucose measurements are not confounded by salivary glucose, which can be influenced by food intake, and salivary glucose concentrations can be monitored for comparison. A study using healthy volunteers who underwent stepwise elevation of blood glucose showed via breath condensate assays that as blood glucose rose, breath glucose concentrations increased, confirming blood glucose directly influences airway glucose levels. The study concluded that blood glucose levels are approximately 12.5 times higher than what is found in the airway in individuals without chronic lung disease [26]. This method indicates that in healthy individuals, blood glucose could be used as a proxy for airway glucose when taking into count the predicted site-dependent differences in concentration.

Another method for airway glucose detection is the use of sputum samples. One study measured glucose in 117 sputum samples from people with CF (pwCF) and found 34% of samples had undetectable glucose, considered under 0.01 mM, while 55% of samples had glucose concentrations above 1 mM. Additionally, over 50 mM glucose was detected in several samples [35]. The use of sputum to determine airway glucose has some complications and limitations. First, the ability to test sputum requires that individuals being evaluated can produce sputum. This can present difficulties in obtaining non-disease controls [34,35]. Individuals with CF on highly effective modulator therapies have also been found to produce sputum samples less frequently, making consistent evaluation of CF sputum more difficult [38]. Another potential complication with sputum glucose measurements is that it is not possible to determine where in the lung the sample originates or how long it has taken the sputum to accumulate over time. Alternatives to sputum include bronchoalveolar lavage (BAL) and nasal lavage (NL) fluid samples [34,39]. An evaluation of BAL and NL samples found significantly lower glucose concentrations when compared with sputum samples from the same subjects, suggesting differences in glucose availability may exist in specific regions of the airway [34].

## 4. Hyperglycemia and Acute Airway Infections

### 4.1. Pneumonia and Hyperglycemia

Incidences and outcomes of community acquired pneumonia (CAP) have been extensively studied across multiple countries, and studies addressing the influence of hyperglycemia on CAP have focused on associations with diabetes. When interpreting outcomes of CAP clinical studies, it is important to recognize that not all studies have the same criteria for diagnosing CAP. Some have diagnostic criteria of either a positive microbiological culture or lower respiratory symptoms and a positive radiological image, while other studies aimed at identifying at the underlying cause of infection required a positive culture. These diagnostic requirements and significant outcomes reported are summarized in Table 2. European studies have found that overall, people with diabetes are at increased risk for hospitalization because of CAP. Specifically, a 3-year Portuguese study using the data from the Ministry of health’s administration found that women with diabetes were more likely to be admitted to the hospital for CAP than women without diabetes (41.5% vs. 38.1%, *p* < 0.0001). The same study also found that both men and women with pneumonia and diabetes had longer hospital stays (12 ± 10.5 days vs. 11.2 ± 10.1 days, *p* < 0.0001) and increased mortality (15.2% vs. 13.5%, *p* < 0.002) compared to those without diabetes [8]. Similarly, a 5-year Spanish prospective study found that pneumonia cases were more severe in diabetics, which correlated with increases in hospitalizations (93% vs. 78%, *p* < 0.001) and mortality (17% vs. 8%, *p* = 0.002) [9]. An 8-year study from Denmark showed people with T1D had an adjusted relative risk of hospitalization due to pneumonia of 4.43, while people with T2D had an adjusted relative risk of 1.23. Men and women with T1D were also found to be at increased risk (adjusted relative risk: male = 3.97; female = 5.28) of hospitalization due to pneumonia compared to those with T2D (adjusted relative risk male = 1.23; female = 1.24) [10]. A Dutch 1-year prospective study found people with T1D or T2D diabetes are at an increased risk for acute lower respiratory tract infections (adjusted odds ratio; T1D = 1.42, T2D = 1.32) but not acute upper respiratory tract infections [11]. Interestingly, it was also observed that the adjusted relative risk of hospitalization was 3.21 in people under 40 with diabetes, while in those 40 and over, the adjusted relative risk was 1.65 [10]. The Copenhagen City Heart study, which included over 10,000 participants, found that individuals who had diagnosed diabetes were found to be at a significantly higher risk for being hospitalized for pneumonia (adjusted hazard ratio 1.75, *p* = 0.002). Having a plasma glucose level at or above 11 mM or diabetes also significantly increased the risk of pneumonia (hazard ratio of 2 and 1.56, respectively). This study also determined that for every 1 mM increase in blood glucose over 11 mM, the risk of pneumonia increased by 6%. However, a significant difference in 28-day mortality with pneumonia between diabetics and non-diabetics was not observed (19% vs. 14%) [12]. An Australian study using a cohort from the Fremantle Diabetes Study found people with T2D are twice as likely to be hospitalized for infection than those without diabetes, with a 1.86 incident rate ratio when hospitalized because of pneumonia [15]. Additionally, a study from Taiwan used 17 years of data collected from the country’s National Health Insurance database and found people with T2D who were on metformin to help manage blood glucose levels had decreased rates of bacterial pneumonia (adjusted hazard ratio of 0.89, *p* < 0.001) and better outcomes, including decreased death (adjusted hazard ratio of 0.64 *p* < 0.001), compared to people with T2D who were not on metformin [40]. These studies highlight how management of diabetes can have important implications for pneumonia outcomes and underscore the need for further studies to examine the differences in T1D and T2D.

### 4.2. Associations Between Airway Pathogens and Hyperglycemia in Pneumonia

The correlation between diabetes and pneumonia is clear; however, few studies draw associations between hyperglycemia and the etiologic agent of pneumonia. Reports of bacterial pathogens in airway diseases that have investigated potential links to hyperglycemia or diabetes are summarized in Table 3. The studies that have analyzed the microbiological causes of pneumonia in individuals with hyperglycemia have provided conflicting results as to what the most prevalent microbial pathogen is. Spanish studies on CAP found that *Streptococcus pneumoniae* was the most common pathogen, while noting that *Staphylococcus aureus* and *Pseudomonas aeruginosa* were also prevalent. Interestingly, there was no difference in the pathogens associated with CAP in diabetic and non-diabetic groups; however, individuals with diabetes had more severe pneumonia [9,41]. High blood glucose upon admission to the hospital with pneumococcal pneumonia was associated with worsening disease severity and worse outcomes for individuals without diagnosed diabetes, especially those who had blood glucose levels above 180 mg/dL (10 mM). The same study also found that people with uncontrolled diabetes were more likely to develop pneumococcal pneumonia [42]. A study from Bangladesh found that *Klebsiella pneumoniae* was the most common cause of pneumonia in their diabetic population, with *S. aureus* and *P. aeruginosa* being the second and third highest causes, respectively [43]. Differences in the most prevalent pathogen observed in independent studies are likely due to regional differences; for instance, *K. pneumonia* has higher CAP rates in healthy populations in Asian countries [44].

Diabetes and hyperglycemia can have a significant impact on pneumonia outcomes in healthcare settings. Two studies conducted in London have shown that hospital patients who have glucose in their bronchial aspirates or who have hyperglycemia are more likely to develop bacterial infections [37,45]. Specifically, intubated ICU patients who had ≥1 mM glucose in their bronchial aspirates had significantly more staphylococcus species present, including significantly more methicillin-resistant *S. aureus* (MRSA). However, no increase in *Pseudomonas*, *Acinetobacter*, or *Enterococcus* species or yeast was observed in the presence of glucose in this patient population [37]. Additionally, patients in critical care who had high blood glucose (at or above 11.1 mM) were found to be more likely to have a sputum sample culture positive for bacterial growth in the first 7 days, compared to those with normal blood glucose levels. In both groups, the most commonly isolated bacterium was a *Pseudomonas* species, with *S. aureus* being the second most common [45]. Differences in pathogen prevalence could be explained by the overall sample size and differences in study design. For example, the study that identified MRSA in ICU patients was conducted at a single hospital, included 98 patients, and stated that screening patients at least twice weekly for MRSA was a normal procedure for their hospital [37]. However, it was unclear if additional testing for other microorganisms was performed simultaneously. This could result in a higher rate of *Staphylococcus* identification compared to other bacterial species. In comparison, the second study included a cohort from two hospitals of 664 patients and only screened for the first 7 days post-hospitalization [45].

Methicillin-resistant *S. aureus* (MRSA) infections are generally considered to be more severe and result in worse outcomes in the context of diabetes. A retrospective observational study from the US found that having MRSA-positive airway cultures increases the risk of mortality in the first 28 days without change in the treatment response [13]. A Chinese study evaluated patients with MRSA pneumonia and found people with diabetes had higher occurrences of MRSA positivity, more severe pneumonia, increased rates of mechanical ventilation, increased mortality, increased antimicrobial-resistant MRSA, and increased co-infections compared to the non-diabetic population. In the diabetic group, the most common bacterial species co-isolated with MRSA was *Acinetobacter baumannii*, while the non-diabetic group was more closely associated with *Klebsiella pneumoniae*. The same study also found that MRSA is a risk factor for mortality regardless of diabetic status [14]. Other studies have shown that glucose is required for *A. baumannii* to form biofilms, allowing the pathogen to persist, and this could explain the increased presence of this pathogen in the diabetic group [46].

In healthcare settings, a US study used a logistic regression model, with data from 5975 patients across 62 hospitals, and found that being female and having diabetes resulted in a low but increased risk of healthcare-associated pneumonia (HCAP) MRSA infection [47]. Interestingly, the antibiotics chosen to treat individuals with MRSA pneumonia and diabetes have been found to influence outcomes. One study found that people with diabetes who received linezolid to treat hospital-acquired MRSA pneumonia had better outcomes than people with diabetes who received vancomycin, while in the nondiabetic control group, there was no difference in outcome between antibiotics [48].

The altered diabetic lung environment is likely to play a role in influencing infection outcomes. There have been some efforts to better define the physiological differences between normal and hyperglycemic airways. To further investigate this question, an in vitro study examining the function of the antimicrobial peptide beta-defensin-2 found the antimicrobial effects against *Escherichia coli*, *P. aeruginosa*, and *S. aureus* were significantly reduced when exposed to high levels of α-dicarbonyls, a common result of hyperglycemia [49]. Another in vitro study found that high glucose conditions inhibit C-type lectin binding to mannose structures, disrupting the immune system’s ability to recognize pathogens [50]. These findings from in vitro studies warrant further confirmation using in vivo models and patient clinical samples. Other than disrupting immune function, there is also evidence that the diabetic environment can be advantageous to the bacteria causing infections. For example, one study found that *S. aureus* formed larger biofilms under hyperglycemic and ketoacidosis conditions than in either condition alone [51]. Additional elements of the altered hyperglycemic lung environment, such as immune dysregulation and altered nutrient availability, may benefit bacterial pathogens. However, these factors are poorly understood and warrant future investigation.

**Table 3:** Bacterial pathogens associated with elevated glucose in chronic airway disease.

Pathogen	Population/Disease Comparison	Outcome
*Pseudomonas aeruginosa*	Diabetic vs. non-diabetic	Equal prevalence in diabetic vs. non-diabetic [41]
	Diabetic only	Third most isolated bacterial pathogen [43]
	Glucose detected in bronchial aspirates vs. not detected	No difference [37]
	Elevated blood glucose	Increase in positive cultures [45]
	Diabetes + COPD vs. COPD alone	Equal prevalence [19]
	CFRD vs. CF	Increased risk of chronic infection [16]
		Increased risk of multi-antibiotic resistance [52]
		Increased prevalence [53]
	Uncontrolled CFRD	Increase in colonization [54]
*Staphylococcus aureus*	Diabetic vs. non-diabetic	Equal prevalence [9,41]
		More likely to be co-isolated with *A. baumannii* [14]
	Diabetic only	Second most isolated bacterial pathogen [43]
	Glucose detected in bronchial aspirates vs. not detected	Increased prevalence [37]
	Elevated blood glucose	Second most isolated bacterial pathogen [45]
	Diabetes + COPD vs. COPD alone	Increased prevalence [19]
	CFRD vs. CF	Increased risk of persistent infection [55]
		Decreased prevalence [56]
	Controlled CFRD	Increase in colonization [53]
*Streptococcus pneumoniae*	Diabetic vs. non-diabetic	Equal prevalence in diabetic and non-diabetic groups [9,41]
	Diabetic only	Sixth most isolated bacterial pathogen [43]
	Uncontrolled vs. controlled diabetes	Increased risk in uncontrolled group [42]
*Klebsiella pneumoniae*	Diabetic only	Most isolated bacterial pathogen [43]
	Elevated blood glucose	Third most isolated bacterial pathogen [45]
*Haemophilus influenzae*	Diabetic vs. non-diabetic	Equal prevalence [9,41]
	Diabetes + COPD vs. COPD alone	Equal prevalence [19]
*Legionella pneumophila*	Diabetic vs. non-diabetic	Equal prevalence [41]
*Escherichia coli*	Diabetic only	Fifth most isolated bacterial pathogen [43]
	Elevated blood glucose	Fourth most isolated bacterial pathogen [45]
*Acinetobacter*	Diabetic only	Fourth most isolated bacterial pathogen [43]
	Glucose detected in bronchial aspirates vs. not detected	No difference [37]
	Elevated blood glucose	Tenth most isolated bacterial pathogen [45]
*Enterococcus*	Glucose detected in bronchial aspirates vs. not detected	No difference [37]
*Stenotrophomonas maltophilia*	Elevated blood glucose	Sixth most isolated bacterial pathogen [45]
	CFRD vs. CF	Increased risk [16]
*Moraxella catarrhalis*	Diabetes + COPD vs. COPD alone	Equal prevalence [19]
*Burkholderia cepacia*	CFRD vs. CF	Increased risk [16]

## 5. Chronic Airway Disease and Hyperglycemia

### 5.1. Chronic Obstructive Pulmonary Disease (COPD)

COPD is a prevalent chronic lung disease that is associated with higher glucose concentrations in the airways. Through analysis of sputum samples, previous studies have shown that people with well-managed COPD have increased sputum glucose concentrations compared to smokers without COPD and non-smokers without COPD, regardless of diabetic status. This study also found that COPD exacerbations result in further increased airway glucose concentrations [34]. Additionally, it has been shown that COPD outcomes are negatively affected by increased blood glucose, with adverse outcomes increasing by 15% and risk of death increasing by 10% for every 1 mM increase in blood glucose [19]. Another study followed people with COPD for 24 months after hospitalization and found that subjects with diabetes had significantly lower survival than those who did not have diabetes [20]. A retrospective study found that individuals who were hospitalized with acute COPD exacerbations and a fasting blood glucose of ≤7 mM had a greater risk of death than those who had lower blood glucose [19]. Another retrospective study observed similar trends, yet once cofounding factors were taken into account, statistically significant differences were not observed [21]. Similarly, a Turkish study found that people with COPD and metabolic syndrome had significantly more exacerbations than those with no metabolic syndrome [22]. Differences in observed outcomes could be due to the consideration of blood glucose level rather than solely diabetic status, or differences in the confounding factors accounted for.

Few studies have analyzed the correlation between lung infections, COPD, and diabetes. However, one study found a significant correlation between having a blood glucose concentration of 7.0 mM or higher and an infection that was polymicrobial or caused by *S. aureus* alone. However, there was no significant increase in other common pathogens known to cause infections in COPD, including *S. pneumoniae*, *Haemophilus influenzae*, *Moraxella catarrhalis*, and *P. aeruginosa* [19].

### 5.2. Cystic Fibrosis

CF is one of the most highly studied chronic airway diseases and is the most common human genetic disease in Caucasian populations. CF disease is the result of mutations in the cystic fibrosis transmembrane regulator (CFTR) gene that lead to a reduction in CFTR protein production or function. Increased glucose concentrations have been observed in the lungs of people with CF (pwCF). Using breath condensate, it has been shown that airway glucose concentration in pwCF can exceed 3 mM, and individuals with CF-related diabetes (CFRD) can have up to 6 mM glucose concentrations in their airways [26]. CFRD is the most common co-morbidity in pwCF, affecting up to 50% of the adult CF population in the US. CFRD is known to negatively correlate with lung function, quality of life, mortality, and overall worsening disease [16,17,57,58]. Individuals with CFTR genotypes associated with worse CF disease are at increased risk of developing CFRD [59,60,61]. Because CFRD is caused by damage to pancreatic beta cells, onset occurs later in childhood and further increases in adolescence and young adulthood [24]. One study monitored the blood glucose levels of people with CF, CFRD, and non-CF non-diabetic controls over the course of 24 hours and found that those with CFRD had significantly higher blood glucose levels over the course of the day than the other two groups [62]. Currently, insulin is the only recommended treatment for those with CFRD [63,64].

Individuals with CFRD and chronic rhinosinusitis reported worse sinus symptoms than pwCF without CFRD, and their symptoms were found to worsen progressively over time [65]. People with CFRD also have worse lung function compared to the nondiabetic CF population [23,66]. Particularly, women with CFRD showed a 20% decrease in lung function and a decreased life span of 17 years compared to women with CF but without a CFRD diagnosis. In males, this difference was only 2 years [18,67]. A more recent study from the UK observed similar results [68]. Other groups observed similar differences between males and females with severe genotypes but not in those with mild disease [59,60].

In CF, it has been observed that as blood glucose levels increase, lung function decreases, as determined by forced expiratory volume in one second (FEV_1_), a measure that approximates lung capacity [69]. Additionally, it has been shown that having an exacerbation or worsening of symptoms, such as cough or sputum production, is correlated with having increased blood glucose and higher glucose tolerance levels [69]. Diabetes and CFRD have also been shown to be significant risk factors for negative outcomes following lung transplant, the only treatment for end-stage disease [70]. Individuals with CRFD are at higher risk to die while waiting on a transplant and have significantly higher mortality and overall shorter survival time when compared to those with pwCF but not CFRD who received a lung transplant [54,70,71]. Despite the negative associations with lung function and outcomes for individuals with CFRD, little is known about the mechanisms that lead to worse symptoms and disease progression. One in vitro study using immortalized CF bronchial epithelial cells showed that potassium and transepithelial chloride currents are dysregulated in hyperglycemic conditions [72]. However, further work in this area is needed to better understand what is taking place at the cellular level during diabetes and hyperglycemia in CF disease.

### 5.3. Infections in CFRD

People with CF are already at increased risk of lung infections, and the pathogens colonizing and infecting the lungs are very well characterized, largely due to the multiple countries publishing annual reports. However, these metrics for people with CFRD are less characterized and show conflicting results. Data from the European Cystic Fibrosis Society Patient Registry (ECFSPR) show that those with CFRD have slightly higher odds of being chronically infected with *P. aeruginosa*, *Burkholderia cepacia*, or *Stenotrophomonas maltophilia*. This study did not discuss any potential differences in *S. aureus* infections [16]. A French study also found that in a small cohort of people with CFRD, individuals with hyperglycemia had significantly more *P. aeruginosa* colonization than those with CFRD with more controlled blood glucose, who trended more towards *S. aureus* colonization [53]. Further data analysis of the Cystic Fibrosis Foundation (CFF) patient registry found that people with CFRD are significantly more likely to culture positive for multiple antibiotic-resistant *P. aeruginosa* and are at increased risk of persistent antibiotic-resistant *S. aureus* infections [52,55]. Alternatively, a small study based in Liverpool, England, found no difference in *P. aeruginosa* or *B. cepacia* colonization regardless of glycemic status [73]. The differences in the outcomes of these studies are mostly likely due to population size. The studies reporting information from the CFF included data obtained from 20,000–30,000 individuals, while the study from Liverpool had a smaller cohort of only 60 people. A more recent study examined the microbiome of people in the UK with CF or CFRD. They found the CFRD lung is more diverse at both the phylum and genus levels when compared to the non-diabetic CF lung. The CFRD data also showed a significant increase in Proteobacteria, mainly *Pseudomonas* species, in individuals with CFRD compared to the non-diabetic CF lung [56]. This increase in *Pseudomonas* may be attributed to decreased lung function commonly observed in CFRD, and decreased lung function is also known to be correlated with *Pseudomonas* infection in pwCF. Supporting this theory, the CFRD cohort in this study had decreased FEV_1_ and FEV_1_/FVC compared to the CF alone cohort.

## 6. Models for Evaluating the Role of Glucose in the Airway

The clinical studies discussed above have clearly demonstrated links between hyperglycemia or diabetes and worse airway disease outcomes. Additionally, analyses of airway surface liquid from chronic airway diseases have shown airway glucose can be elevated in these patient populations, which may potentiate more frequent and worse bacterial respiratory infections. Accurately modeling the airway environment is critical to investigating factors in the hyperglycemic lung that may lead to increased infections. Three major model systems have been used for laboratory studies to evaluate how hyperglycemia affects the lungs and airway epithelium. These include airway cell culture, mouse models, and artificial sputum media. Here, we discuss methodology for the use of these systems and data obtained from studies in these models, as well as the potential for future studies that will allow us to better define the relationships between bacterial pathogens and elevated glucose in airway environments.

### 6.1. Airway Cell Culture Models

The lung environment is commonly modeled in vitro using airway cell culture. Primary and immortalized bronchial epithelial cells from humans or other mammals are frequently used to model the environment in the large airways, and many cell lines have been tested in hyperglycemic conditions. Human immortalized cell lines can be used in a submerged culture where the cells are in direct contact with cell culture media for the duration of the experiment. If used for infection studies, this also means any bacteria used in submerged culture models are also exposed to any available nutrients and other factors present in cell culture media; otherwise, complete media may be removed from cells, resulting in transient starvation. Airway cells can alternatively be cultured on a permeable filter in transwell systems to establish an air–liquid interface (ALI), where cells are fed from a basal chamber with an air-exposed apical surface. ALI culture mimics the lung environment more accurately for infection studies by creating a barrier between the nutrient-rich environment of the cell culture media and nutrients the bacteria encounter at the cell surface. Different cell lines or airway cell types have specific recommended media for maintenance in culture, and glucose concentrations can vary in base culture media. Primary airway cells often require different media for the expansion and maintenance of ALI cultures, and media recipes can be complex, requiring many defined additives, or recipes may be proprietary depending upon information provided by vendors. These factors can make it difficult to adjust media for primary cell culture to test the effects of high glucose. The benefits of using immortalized cell lines rather than primary human cells include the ease of altering the culture media to adjust glucose concentrations to specific amounts that mimic those observed in the bloodstream, without relying on consistent human donors.

Numerous immortalized airway cell lines have been used to study the CF and non-CF airway epithelium and address questions regarding the role of high glucose in airway function. One common immortalized non-CF cell line used is the A549 cell line that was originally isolated from human alveolar cells from an adult male with carcinoma. A549 cells were cultured at ALI with 0-, 10-, 20-, or 50-mM glucose in the basolateral media and then infected with *P. aeruginosa*, strain PAO1, for 4 hours. This study observed that *P. aeruginosa* had increased burden at the most highly elevated 20 and 50 mM glucose concentrations, but not 10 mM, when compared to the 0 mM glucose condition [45,74]. However, a disadvantage of the A549 cell line is that ALI cultures have been observed to have very low transepithelial electrical resistance (TEER), a measure of epithelial barrier integrity [75,76].

NCI-H441 cells are also alveolar cells that were isolated from an adult male without CF disease with lung cancer [77]. These cells can form tight junctions, they have polarized monolayers, and produced good TEER measurements supporting intact barrier function in ALI culture [78]. These cells have been used study the effects of glucose on *S. aureus* infections. It has been shown that *S. aureus* exhibited increased growth when co-cultured with cells fed with 20 mM glucose compared to 5 mM or 10 mM [79,80]. However, to observe increased growth for *P. aeruginosa*, the basolateral glucose had to be increased to 40 mM compared to 10 mM [79]. The researchers also found that pretreating cells with 1 mM metformin to control apical glucose concentrations decreased the *S. aureus* burden [79,80]. Other studies that pretreated H441 cells with metformin found that it prevented *S. aureus* from disrupting occludin and zonula occludens proteins [81].

Another lung non-CF cancer cell line commonly used in laboratory research is the Calu-3 cell line. These cells form tight junctions and express CFTR [82]. Studies using these cells have found that *P. aeruginosa* grew better when co-cultured on cells grown with 15 mM glucose compared to 5 mM. The same study also found that treating the cells with metformin before *P. aeruginosa* inoculation reduced bacterial burden and slightly increased TEER during infection. However, metformin pre-treatment did not restore TEER to uninfected levels, although it did prevent the loss of the tight junction protein claudin [83].

Two commonly used bronchial epithelial cells lines for making non-CF and CF comparisons are the 16HBE14o- and CFBE41o- cell lines. 16HBE14o- cells were originally derived from the bronchial cells of an adolescent male [84]. These cells retain tight junctions and ion transport. In a study where 16HBE14o- cells were cultured at ALI with 5.5- or 17.5 mM glucose in the basolateral media, it was observed that hyperglycemia increased CFTR current and did not cause changes to the expression of tight junction proteins claudin or zonula occludens in cells with normal CFTR function [85]. CFBE41o- cells were isolated from the trachea of a 21-year-old male with CF homozygous for the ΔF508 mutation [86]. These cells can be transduced with WT-CFTR to restore function [87]. CFBE41o- cells have been used to determine the effects of high glucose (25 mM) on CFTR function and wound healing. Here, it was observed that cells maintained in the high-glucose condition had reduced CFTR function compared to the normal (5 mM) glucose condition. The same study also found that the high-glucose condition significantly reduced wound healing, as determined by a scratch assay [72]. Similarly, the effects of hyperglycemia have also been investigated in 16HBE14o- cells expressing a modified CFTR, F508del/V470. Using these cells, it was found that treating cells with modulator therapy improved CFTR current under both normal and hyperglycemic conditions. The same study also observed that hyperglycemia increased both claudin and zonula occludens expression compared to normal glucose. Treatment with modulator therapy decreased claudin expression in the hyperglycemic condition, while increasing zonula occludens expression [85].

Primary bronchial cells are isolated directly from the lung tissue and are the closest cells to the lung environment. Primary cells are best used with minimal passaging, but they have been passaged one to two times and retained appropriate cellular functions [88]. This limits the experiments that can be conducted with primary cells, as there must be tissue available. Another limitation to using primary cells to determine hyperglycemic effects is that most base medias already contain high concentrations of glucose. Primary bronchial cells have been used to study glucose uptake and glucose flux across the epithelial layer, including host glucose transporters [27,28]. Additionally, other researchers have used both primary bronchial cells and primary CF bronchial cells to study the difference between normal and hyperglycemic conditions. They found that in both cell types, hyperglycemia increase proinflammatory mRNA levels and reduces voltage-dependent K+. In CF cells, they also found that hyperglycemia significantly decreased ASL volume [89]. Other studies with primary cells have shown that glucose is required for *P. aeruginosa* to grow in ASL [27].

Recently, a methods paper was published using automated cell culturing to better mimic the fluctuating blood glucose levels seen in those with CFRD [90]. Other recent efforts to improve upon traditional cell culture have included more advanced models that incorporate multiple cell types to better recapitulate the human airways, such as lung-on-a-chip and organoid culture systems [91,92]. Further, advances in techniques such as single-cell signaling have yielded important data on the functions of distinct cell types within the epithelium and have even identified novel cell types. For example, recent work has characterized ionocytes and their relationship to CFTR expression and function [93,94]. How high-glucose exposure affects epithelial cell functions has yet to be investigated in these newer systems or with respect to specific cellular subtypes, and further development of these models could provide novel insights regarding relationships between hyperglycemia and infection in CF and chronic airway disease.

### 6.2. Animal Models

Mice are the most used animal for modeling diabetic airway infections. Mice can either be bred to develop diabetes or have diabetes artificially induced via chemical treatment. To induce diabetes, animals are injected with streptozotocin, a drug that induces diabetes by causing pancreatic β cell death. Within 3 days, treated mice will develop hyperglycemia. This treatment allows multiple backgrounds of mice to be used without other complicating side effects like obesity, and it enables the evaluation of disease backgrounds, such as CF mice. Genetically, obese (ob/ob) mice that are leptin-deficient are often used as a prediabetic model. Diabetic (db/db) mice are distinct from obese mice and harbor a mutation in the leptin receptor gene resulting in hyperglycemia and obesity. More recently, a transgenic mouse diabetes model has been developed. These mice develop mild diabetes due to impaired insulin secretion caused by disruption in pancreatic β cells, without the obesity or leptin deficiencies other genetic mouse models have been found to develop [95].

Diabetic mouse models have been used to further investigate the outcomes of bacterial respiratory infections with traditional CF pathogens. A study using obese (ob/ob) and obese diabetic (db/db) mice found that they failed to clear *P. aeruginosa* lung infections compared to the littermate controls. However, the mice were able to clear a *P. aeruginosa* mutant that could not use glucose [27]. A similar study using obese diabetic (db/db) mice found that mice had higher blood glucose, bronchoalveolar lavage fluid (BALF) glucose, white blood cells, and neutrophil levels compared to WT (C57BL/6) mice, as well as increased *P. aeruginosa* CFUs in BALF [39]. Other studies have found obese diabetic (db/db) mice have higher *S. aureus* lung CFU burden compared to WT mice. Interestingly, this study also found that treating the diabetic mice with metformin reduced CFU levels but did not affect the blood glucose levels of the mice [79]. Similar results have been shown in C57BL/6 mice injected with streptozotocin; metformin had no effect on blood glucose levels but did reduce *P. aeruginosa* BALF CFU burden to WT levels [45].

A limited number of studies have investigated the effects of CFRD in mice. The first group to address this question used streptozotocin to induce diabetes in transgenic mice that harbored the FABP-hCFTR transgene, where the intestinal promoter for rat fatty acid binding protein 2 directed expression of a human CFTR gene, and a targeted knock-out mutation of the murine CFTR homolog gene (C*ftr^tm1Unc^* Tg(FABPCFTR)-1Jaw/J). They observed that these animals had the expected elevated blood and BAFL glucose levels compared to CF non-diabetic mice. When challenged with *P. aeruginosa* infection, the CF diabetic mice had significantly higher bacterial burden and increased BALF leukocytes, macrophages, and granulocytes [96]. Another group recently established a CFRD mouse model using Scnn1b-Tg mice, which develop CF-like lung disease, and induced diabetes with streptozotocin. This model also produced the expected increases in blood and BALF glucose levels, and an increase in overall inflammatory cell numbers, including lymphocytes, was observed when compared to nondiabetic mice [97].

### 6.3. Artificial Sputum Medium

Artificial sputum media, or ASM, is defined media that nutritionally mimics the airway environment and may include additional extracellular polymers, host proteins, and vitamins to recapitulate airway surface liquid in chronic disease. Defining an ASM recipe has traditionally required first collecting sputum samples from individuals with CF. Samples may then be lyophilized and subjected to analytical chromatography to determine the concentrations of anions, cations, and amino acids present [36]. Concentrations of carbon sources, including glucose and lactate, can be measured using commercially available kits or mass spectrometry analysis. There are many different versions of ASM, which were compared in detail by Neve et al. in 2021 [98]. Since then, new recipes have been developed, including for the sinuses [99].

The defined nature of ASM allows for the media to be adjusted to accurately represent glucose levels that would be present in the lungs of individuals with CF or other airway diseases. Glucose concentrations in commonly used ASM recipes typically range from 0–3 mM [36,99,100,101,102]. However, the ASM recipes published to date have not accounted for subjects’ blood glucose levels at the time of sputum sample collection and have not described the CFRD statuses of donors. While the use of glucose as a carbon source in other defined lab media has been extensively studied for many species of bacteria, including traditional CF pathogens, surprisingly few published studies have compared how adjusting glucose in ASM changes bacterial growth [36,103,104]. One study tested bacterial growth in ASM with or without 4 mM glucose and found that when *S. aureus* and *P. aeruginosa* were cultured separately, there was no difference in final bacterial burden in media with or without glucose. However, when the two bacteria were co-cultured, *S. aureus* grew significantly better in the presence of *P. aeruginosa* in ASM with glucose, while *P. aeruginosa* showed no change [105]. This suggests glucose may affect interspecies competition, benefiting some species over others in cases of polymicrobial infections.

## 7. The New Era of Highly Effective Modulator Therapies: What We Know About CFTR Modulators, Glucose, and Infection

CFTR modulator therapy has significantly improved many aspects of CF disease. Despite this, there have not been as significant improvements observed in the CFRD status of individuals receiving highly effective modulator therapies (HEMTs) as was initially hoped. Ivacaftor was the first CFTR modulator approved by the FDA in 2012 [106]. After 5 years of ivacaftor approval, the number of patients in the US with CFRD was reduced by 5.2% compared to those not receiving ivacaftor. In the UK, this difference was 10.5% [107]. Another study used data from the US CF Foundation Patient Registry and found that people on ivacaftor who also had CFRD still experienced greater declines in lung function over time compared to those without CFRD [108]. To further improve CFTR function, there is now a triple corrector therapy (elexacaftor/tezacaftor/ivacaftor, or ETI) in use. Despite improved lung function 2 years after beginning ETI treatment, only slight improvements in hemoglobin A1c and fasting glucose were observed in study groups [109].

HEMTs have been found to greatly improve lung infection status in many people with CF. However, trials evaluating ivacaftor alone, as well as ETI, have found that while there is a reduction in *P. aeruginosa* and *S. aureus* burden within the first month of starting treatment, bacteria still maintained persistent and detectable levels even after years of treatment [107,110,111]. These data highlight the remaining efforts required to advance understanding of the effects of CFRD on lung physiology and its impact on infections. Gaining a more comprehensive understanding of the effects of hyperglycemia and diabetes on the CF airway environment, and how HEMTs affect CFRD status over time in young and aging CF populations, may yield new therapeutic approaches that can address persistent infections and lead to improvements in the eradication of bacterial pathogens in people with CF.

## 8. Conclusions

The diabetic lung provides a unique environment for bacterial infections that is not fully understood. Clinically, only glucose levels have been evaluated and found to be altered in diabetic lungs; however, it is still unknown whether this alone is enough to increase infection with specific airway pathogens, or whether other cellular processes are being affected due to the high-glucose environment. For example, if the innate immune response to infection is less effective in the presence of elevated airway glucose, or if inflammation is exaggerated, this may alter the ability of the airway to properly clear invading bacteria or result in increased tissue damage. While some studies have shown increased respiratory pathogen growth in diabetic cell culture and animal models, to date, this work has mainly focused on measuring overall bacterial burden and general markers to approximate the host immune response. To our knowledge, no studies have examined whether bacterial responses to therapeutics are affected under high-glucose conditions in the lungs. The lack of information available regarding the effectiveness of antibiotics and other lung disease therapeutics in individuals with diabetes or hyperglycemia is a major gap in knowledge. Determining if bacteria have an altered response to antibiotics under high-glucose conditions could greatly improve infection outcomes. The use of the model systems discussed above to consider the impacts of high glucose on infection in airway environments may yield crucial data to improve treatment effectiveness in the coming years. Additionally, such research would have broader beneficial effects for other patient populations that are known to have glucose present in their bronchial aspirates, in addition to people with diabetes.

## Figures and Tables

**Table 2:** Summary of clinical studies on pneumonia and hyperglycemia.

Study Location	Study Size	Pneumonia Diagnostic Criteria	Major Outcomes
Portugal [8]	Total: 74,175 Diabetes: 19,212	Chart code with CAP the main reason for admission (ICD-9-CM 480–486, 487)	Age Sex Length of hospital stay Mortality
Spain [9]	Total: 660 Diabetes: 106	Acute lower respiratory tract infection with radiological consolidation in chest	Hospitalization Age Comorbidities Pleural effusion Mortality Etiologic agents
Denmark [10]	* Total: 349,854 Diabetes: 32,975 T1D: 288 T2D: 32,687	Discharge diagnosis of pneumonia, legionellosis, or ornithosis (ICD-10 J12.x-J18.x; A481x; A70.x)	Hospitalization Time since diabetes diagnosis A1C levels Comorbidities
Netherlands [11]	Total: 26,328 Diabetes: 7417 T1D: 705 T2D: 6712	ICPC code R81	Incidences of infection
Denmark [12]	Total: 10,063 Diabetes: 353	Danish National Hospital Discharge Register (ICD-8 and ICD-10 480–486, A48.1, J18)	Hospitalization Type of infection Plasma glucose levels Mortality after hospitalization
Australia [15]	Total: 5156 T2D: 1289	Chart admission code for pneumonia (ICD-9-CM 480.1, 480.2 480.8, 480.9, 481, 482.0–482.9, 483.0, 485, 486; ICD-10-AM J12.1, J12.2, J12.8, J12.9, J13, J14, J15.0, J15.1, J15.3, J15.4, J15.5, J15.6, J15.7, J15.8, J15.9, J18.0, J18.8, J18.9)	Hospitalization Incidence of infection Systolic blood pressure Serum triglycerides Ischemic heart disease Aboriginal racial background Prior hospitalization for any infection
Taiwan [40]	T2D: 98,024 Metformin: 49,012	Chart review based on the Taiwan Bureau of National Health Insurance (ICD-9-CM pneumonia 480–486 or bacterial pneumonia 481, 482.41, 482.8, 486)	Hospitalization Noninvasive ventilation Invasive ventilation Respiratory cause of death

* *Ten control subjects were randomly selected based on birth year, sex, and residence in the same country*.
